# Fluoroquinolone-Mediated Inhibition of Cell Growth, S-G_2_/M Cell Cycle Arrest, and Apoptosis in Canine Osteosarcoma Cell Lines

**DOI:** 10.1371/journal.pone.0042960

**Published:** 2012-08-21

**Authors:** Kyoung won Seo, Roseline Holt, Yong-Sam Jung, Carlos O. Rodriguez, Xinbin Chen, Robert B. Rebhun

**Affiliations:** 1 Department of Surgical and Radiological Sciences, University of California Davis School of Veterinary Medicine, Davis, California, United States of America; 2 Department of Internal Medicine, College of Veterinary Medicine, Chungnam National University, Daejeon, Republic of Korea; Bauer Research Foundation, United States of America

## Abstract

Despite significant advancements in osteosarcoma research, the overall survival of canine and human osteosarcoma patients has remained essentially static over the past 2 decades. Post-operative limb-spare infection has been associated with improved survival in both species, yet a mechanism for improved survival has not been clearly established. Given that the majority of canine osteosarcoma patients experiencing post-operative infections were treated with fluoroquinolone antibiotics, we hypothesized that fluoroquinolone antibiotics might directly inhibit the survival and proliferation of canine osteosarcoma cells. Ciprofloxacin or enrofloxacin were found to inhibit p21^WAF1^ expression resulting in decreased proliferation and increased S-G_2_/M accumulation. Furthermore, fluoroquinolone exposure induced apoptosis of canine osteosarcoma cells as demonstrated by cleavage of caspase-3 and PARP, and activation of caspase-3/7. These results support further studies examining the potential impact of quinolones on survival and proliferation of osteosarcoma.

## Introduction

Osteosarcoma (OSA) represents the most common primary bone tumor in pet dogs. Surgical amputation of appendicular OSA serves to remove the primary tumor and alleviate tumor-associated pain but, unfortunately, the vast majority of dogs harbor occult metastasis at the time of diagnosis [1], [2]. As an alternative to amputation, limb-salvage procedures can provide adequate local control and yield median survival times similar to those reported in dogs undergoing amputation. While post-operative limb-spare infections are quite common in the dog, multiple studies have found such infections to be associated with improved survival when compared with similarly treated dogs without infection [3]–[5]. One such study reported that 24 out of 32 dogs experiencing post-operative allograft infections were treated with fluoroquinolone antibiotics [3], a class of drugs known to have independent activity against several tissue and cancer cell lines *in vitro* and *in vivo* [6]–[8]. Fluoroquinolones (FQs), such as ciprofloxacin (CPFX) and enrofloxacin (ENFX), target topoisomerase enzymes and in this way share a similar mechanism to traditional anti-neoplastic agents such as doxorubicin, irinotecan, and etoposide [6], [7], [9]. Some FQs also have the ability to reverse multidrug resistance-associated protein mediated drug resistance [10], promote microRNA processing [11], induce immunomodulatory effects [12], and are cytotoxic to vascular endothelial cells [13]; indicating that they may possess both direct and indirect anti-tumor activity when used in combination with traditional chemotherapy.

Based upon these previous clinical and in vitro studies, we questioned whether CPFX or ENFX might have direct effects on canine osteosarcoma cells. We hypothesized that these specific quinolones could directly inhibit the growth and/or survival of canine OSA cells in vitro. To test this hypothesis, we exposed canine OSA cell lines to CPFX or ENFX and performed in vitro measures of proliferation and survival. Furthermore, we examined CPFX and ENFX-induced changes in expression of proteins associated with cell cycle progression and apoptosis in canine OSA cells.

## Results

### Ciprofloxacin or enrofloxacin decreases total viable cell number of canine OSA cells

**Figure 1:**
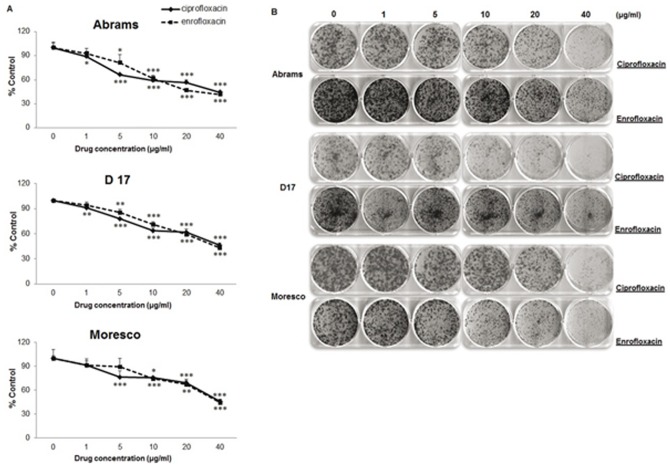
The inhibitory effects of CPFX or ENFX on cell proliferation and colony formation in canine OSA cell lines (Abrams, D17, Moresco). **A.** Inhibitory effect of CPFX and ENFX on cell proliferation evaluated by MTS assay. Data are presented as percent control. **B.** Influence of canine OSA cells on the number of colony-forming cells treated with CPFX or ENFX was evaluated by clonogenic assay. Representative dishes by colony-forming assay. Error bars represent S.D. Statistical significance was assessed using one-way ANOVA with post-hoc Tukey's testing and expressed as follows: * *p*<0.05, ** *p*<0.01, *** *p*<0.001.

We investigated the effect of CPFX or ENFX on three different canine OSA cell lines. A significant decrease in the number of live tumor cells was observed when OSA cells were exposed to concentrations exceeding 1 µg/ml of CPFX in Abrams and D17 and 5 µg/ml in Moresco cells. Concentrations exceeding 5 µg/ml of ENFX in Abrams and D17, and 10 µg/ml in Moresco cells (Figure 1A; *p*<0.05) were required to inhibit the number of live cells measured at day 4. CPFX or ENFX also inhibited colony formation in all three canine OSA cell lines in a concentration dependent manner (Figure 1B).

### Ciprofloxacin or enrofloxacin induces S-G_2_/M arrest

**Figure 2:**
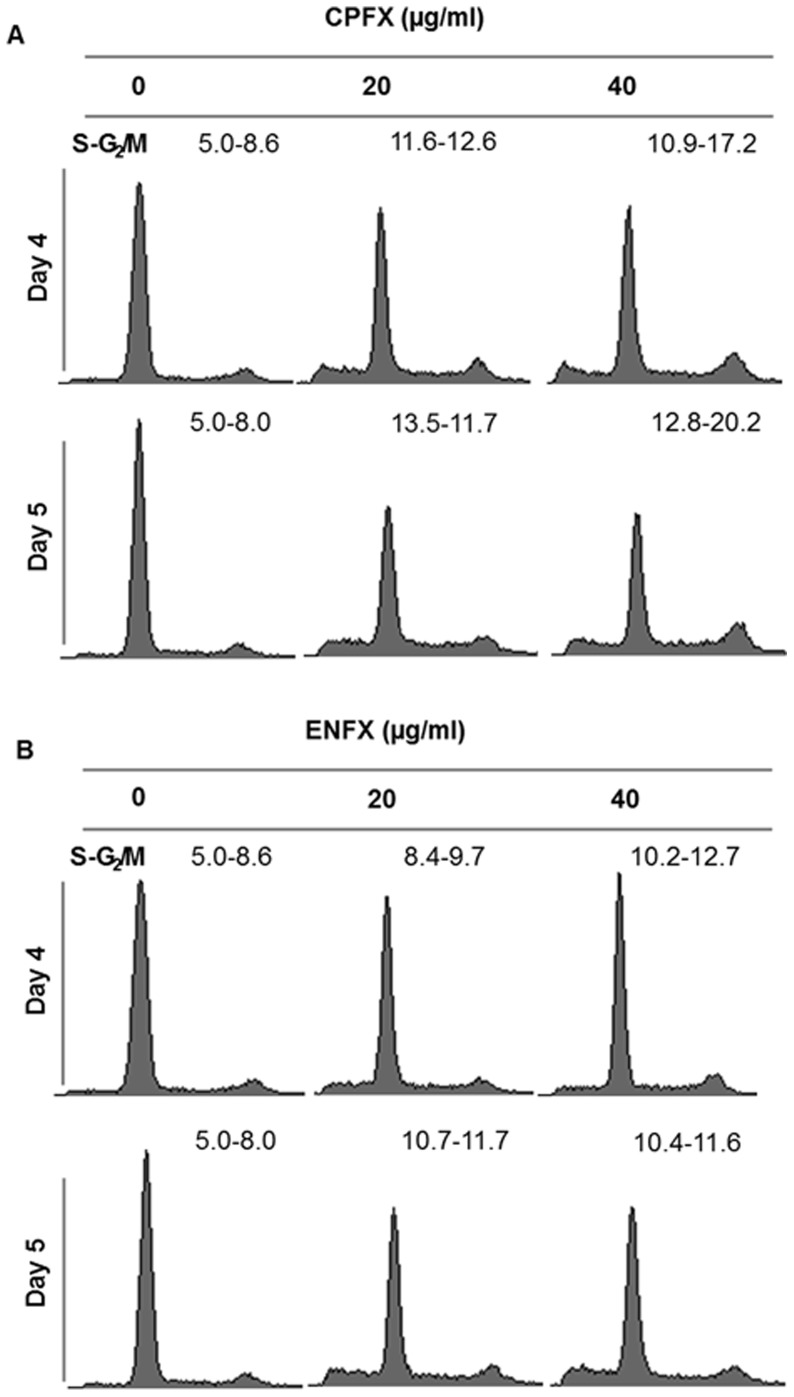
CPFX and ENFX -induced S-G_2_/M arrest on in canine OSA cells. The effect of CPFX (**A**) or ENFX (**B**) on cell cycle distribution was investigated by flow cytometry in Abrams cells following 4 days or 5 days of treatment. Decreases in the proportions of cells in the G_1_ phase of the cell cycle and increases in the S and G_2_ phases were observed. The actual percentage of gated cells are presented in **Table 1**.

Based on the similarity in sensitivity between canine OSA cell lines, we chose to focus mechanistic studies on Abrams cells. The Abrams cell line seemed ideal based on previously published positive characteristics of this cell line including in vivo growth and the ability to form pulmonary metastases in nude mice [14]. In order to explore the mechanism of FQ induced inhibition of OSA cells, we performed cell cycle analyses. CPFX and ENFX treatment of Abrams cells resulted in S-G_2_/M phase cell cycle accumulation after exposure for 4 and 5 days at concentrations at or above 20 ug/ml (Table 1). In addition, CPFX or ENFX both increased the number of canine osteosarcoma cells in sub G_1_ (Figure 2).

**Table 1:** Percentage of gated cells in cell cycle analysis. CPFX and ENFX treated with 20 or 40 ug/ml for 4 or 5 days on Abram cell.

	Drugs	Dosage ((µg/ml)	<g_1_ (%)	G_1_ (%)	S (%)	G_2_/M (%)
Day 4	Control	0	2	82.5	5.0	8.6
	CPFX	20	13.1	60.7	11.6	12.6
		40	11.5	58.1	10.9	17.2
	ENFX	20	9.5	70.9	8.4	9.7
		40	6.6	69.2	10.2	12.7
Day 5	Control	0	1.7	83.8	5.0	8.0
	CPFX	20	13.6	59.2	13.5	11.7
		40	11.3	52.1	12.8	20.2
	ENFX	20	13.1	62.8	10.7	11.7
		40	14.0	62.3	10.4	11.6

### Altered expression of cdc2 and phosphorylated cdc25C in ciprofloxacin or enrofloxacin treated canine Abrams OSA cells

**Figure 3:**
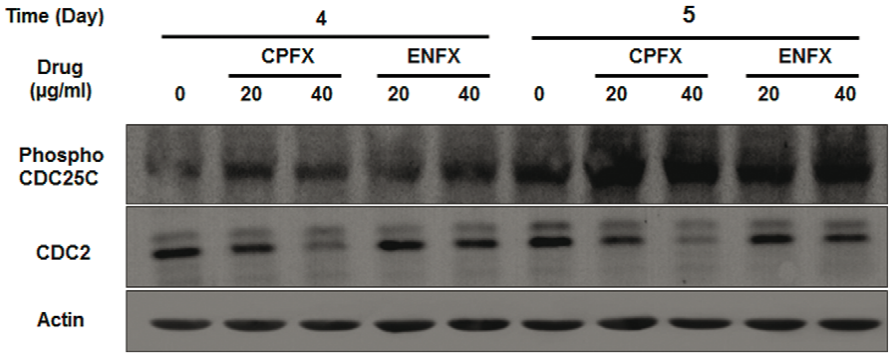
G_2_ checkpoint kinases affected by CPFX and ENFX treatment. Western blot analysis of cdc2 (CDK1) and cdc25C in Abrams OSA cells at day 4 and 5. Both CPFX and ENFX inhibit G_2_ checkpoint kinase cdc2 although CPFX showed more potent than ENFX. The level of phospho-cdc25C was increased in both CPFX and ENFX treated canine Abrams OSA cells.

To further examine the mechanism behind S-G_2_/M phase cell cycle accumulation we examined the effects of FQ's on expression of several checkpoint proteins by western blot analysis. Treatment with CPFX at 20 or 40 µg/ml resulted in reduced protein expression of cdc2 (34 kDa) at five days, whereas reduced protein expression at 4 days was only apparent at the 40 µg/ml concentration (Figure 3). Consistent with results of the cell cycle analysis, while ENFX appeared less potent than CPFX, it was still effective at reducing the cdc2 expression in canine OSA cells; most notable at a concentration of 40 µg/ml on day 5 (Figure 3). In contrast, ENFX or CPFX resulted in similar increases in expression of phospho-cdc25C in canine OSA cells after 4 or 5 days of exposure.

### Ciprofloxacin and enrofloxacin activate caspase- 3 and 7

**Figure 4:**
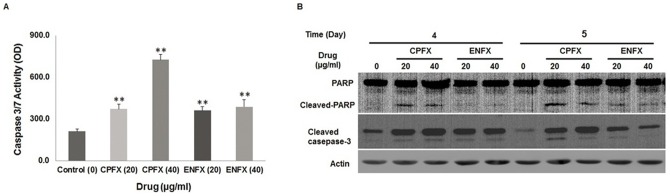
Activation of apoptotic cell death induced by CPFX or ENFX. **A.** Caspase-3/7 activity was measured in relative fluorescence unit with a commercial assay kit in canine Abrams OSA cells following incubation with CPFX (20 and 40 µg/ml) or ENFX (20 and 40 µg/ml) for 72 hours. ** Significantly (*p*<0.01) different from value for untreated control cells. **B.** Western blot analysis of PARP cleavage and cleaved caspase-3. Apoptosis is indicated by the increase of cleaved-PARP and cleaved-caspase-3.

Because OSA cell exposure to FQ's resulted in an increased sub G_1_ fraction on cell cycle analysis, we wanted to examine whether FQ's were specifically capable of inducing apoptosis. We found that Caspase-3/7 activity increased significantly (*p* = 0.005, 0.001, 0.008, 0.002) in canine Abrams OSA cells after incubation either with 20 or 40 µg/ml of CPFX or ENFX (Figure 4A). CPFX induced activation of caspases was dose-dependent (*p* = 0.001), whereas no significant difference was detected between 40 µg/ml of ENFX and the 20 µg/ml concentration (*p* = 0.684).

### Ciprofloxacin and enrofloxacin induce apoptosis

In order to further confirm that FQ's were capable of inducing apoptosis we performed Western blot analysis of PARP cleavage which showed an increase in the cleaved fragment (89 kDa) in Abrams cells treated with 20 or 40 µg/ml of CPFX at day 4, whereas, both ENFX and CPFX increased PARP cleavage at day 5 (Figure 4B). Similarly, cleaved caspase-3 protein (cleaved to yield 17 and 19 kDa) was found to be increased by western blot analysis when compared with untreated control cells at day 4 and 5. Collectively, CPFX and ENFX were both found to induce apoptotic cell death in canine OSA cells.

### Ciprofloxacin and enrofloxacin reduce expression of p21 ^WAF1^

**Figure 5:**
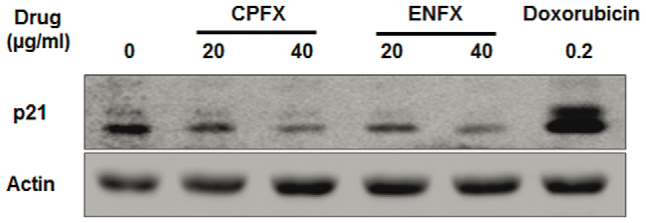
Decreased expression of p21 by CPFX or ENFX. Western blot analysis in Abrams OSA cells treated with CPFX or ENFX at a dose of 20 µg/ml or 40 µg/ml at day 2. Abrams cells treated with doxorubicin were used as a positive control.

Lastly, to further investigate the mechanism of FQ induced changes in proliferation and survival we examined the effects of FQ's on p21 expression by Western blot. We found that expression of p21 in canine Abrams OSA cells was markedly reduced after 48 hour exposure to either CPFX or ENFX (Figure 5).

## Discussion

While adjuvant chemotherapy is known to improve the disease-free interval and median survival time in canine and human patients treated with adequate surgical control, survival has remained relatively static for the past 20 years. However, post-operative limb-spare infections have been associated with improved survival in both species [3]–[5], [15]. While some evidence supports the notion that infection and/or a systemic anti-tumor immune response can inhibit growth of canine OSA [16]–[20], one clinical report noted that the majority of dogs experiencing post-operative infection were treated with FQ antibiotics [3]. We therefore questioned whether FQ's might have direct effects on canine OSA cells and set out to investigate any direct effects of commonly used FQ's on canine OSA cell lines in vitro.

ENFX is currently approved for use in several domestic species, and is commonly prescribed in veterinary species with susceptible infections. ENFX differs from CPFX by the presence of an ethyl group, but pharmacokinetic elimination of ENFX is partially accomplished by means of hepatic metabolism to CPFX [21], [22]. Canine tissue levels of ENFX and CPFX vary significantly between tissues and are dependent on the tissue type and the administered dose [23], [24]. In inflammatory tissues, the concentrations of ENFX and CPFX have been measured as high as 57 µg/g and 6 µg/g, respectively, after administration of a single 20 mg/kg dose of ENFX [24]. At this same administered dose, mean tissue concentrations of ENFX and CPFX after a single dose of ENFX reach 18.7 and 2.8 µg/g in the lung, respectively, [23] which are within the range of concentrations used in this study. However, it should be cautioned that these measured tissue concentrations are based on tissue homogenates and may not be truly representative of concentrations to which OSA cells would be exposed in vivo. We evaluated the effect of ENFX or CPFX in concentrations ranging from 1–40 µg/ml of the drug and found dose and time-dependent effects (Figure 1). We have further determined that similar levels of CPFX or ENFX can alter expression of cell cycle proteins and induce apoptosis of canine OSA cells.

Several studies have demonstrated the cytotoxic and apoptosis-inducing effects of FQ antibiotics on tendon cells, chondrocytes, and a variety of human cancer cell lines [8], [25]–[29]. However, previous studies used a wide range of concentrations, and multiple mechanisms of action have been implicated. The accumulation of Abrams cells treated with ENFX or CPFX in the S and G_2_/M phase of the cell cycle with concomitant induction of apoptotic cell death is in agreement with previous reports of topoisomerase II inhibition by CPFX resulting in S-G_2_/M arrest of prostate carcinoma, transitional cell carcinoma, and colorectal carcinoma cells [25], [28], [29]. While cell cycle analysis and apoptotic death were not specifically evaluated in one previous study using human OSA cells, our findings are consistent with previous reported results demonstrating that CPFX is capable of inhibiting the growth of human OSA cells at concentrations as low as 20 µg/ml [6].

Furthermore, we found that CPFX and ENFX decreased cdc2 expression concomitant with an increase in expression of phospho-cdc25C and directly determined an S-G_2_/M cell cycle arrest by flow cytometry (Figure 2, 3). Decreased expression of cyclin-dependent kinase 1 (cdc2) has previously been reported in rat tendon cells treated with 50 µg/ml of CPFX [30]. Regulation of cdc2-B1 complex involves and activating phosphate by CDK-activating enzyme and inhibitory phosphates at a pair of amino acids in the roof of the active site by Wee 1. Dephosphorylation of these sites by phosphatase cdc25C results in increased CDK activity [31]. DNA damage is associated with many cellular events, including activation of CHK1, which in turn phosphorylates and inactivates cdc25C, allowing inactivation of the 2-B1 complex and G_2_/M arrest [32].

CPFX and ENFX also led to decreased expression of the cyclin dependent kinase inhibitor p21^WAF1^ (Figure 5). Previous studies have also demonstrated p21^WAF1^ down-regulation in CPFX treated cancer cells and indicated that the observed down-regulation of p21^WAF1^ was likely due to the degradation of p21^WAF1^ following ubiquitination by the 26S proteasome-mediated degradation pathway [28]; and that the observed degradation was closely correlated with the time of induction of apoptotic cell death [29]. Inhibition of p21^WAF1^ has previously been shown to sensitize ME-180 OSA cells and MG63 human OSA cells during TNF or anti-Fas-induced apoptosis [33], [34]. This strongly suggests the need to further evaluate the effect of FQs on mitochondria as well as the proteasome-ubiquitination system in canine cancer cells and raises the possibility that FQ's could possibly enhance host mediated immunologic cytotoxicity of OSA.

In addition to cell cycle perturbations, our experiments revealed an in vitro apoptotic effect of CPFX or ENFX on canine OSA cells, in a dose - and time -dependent manner. Caspase-3 is a key member of the caspase family, which is the central component of the apoptotic machinery during apoptotic cell death. Poly (ADP-ribose) polymerase (PARP) is a common death substrate for activated enzymes of caspase family and caspase-3 and caspase-7 demonstrated both cleave PARP [35]. As shown in Figure 4B, activation of caspase-3 after ENFX or CPFX treatment of the canine OSA cells was confirmed by western blot analysis and the activation of caspase-3 was correlated with the proteolytic cleavage of PARP. These results correspond with previous investigations in human bladder cancer cells exposed to 100–300 µg/ml of CPFX [28].

The experiments in this study were designed to determine whether or not FQs were directly capable of inhibiting survival and proliferation in canine OSA cells. Based on our findings, it is possible that the use of FQ's in clinical patients may have contributed to the improved outcome of dogs with OSA. However, much work is needed before such a conclusion can be made. While our results support further study of FQs or possibly other quinolones in the treatment of OSA, many questions remain. Specifically of concern is that while reduction in p21 has been previously shown to increase apoptosis in some cells, loss of p21 expression can increase cellular proliferation in other cells and may contribute to carcinogenesis and tumor progression [36]. Therefore, our results must be interpreted with caution until further in vitro and in vivo studies can be performed.

## Materials and Methods

### Drugs

CPFX (catalogue #17850) and ENFX (catalogue #17850) were purchased from Sigma and dissolved in 0.1N HCL (pH = 1) at a concentration of 25 mg/ml or 10 mg/ml respectively.

### Cell cultures

Abrams and Moresco cell lines were kindly provided by Dr. Douglas Thamm, Department of Clinical Sciences College of Veterinary Medicine and Biomedical Sciences Colorado State University. D-17 (CCL-183) cell was purchased from American Type Culture Collection. Cell lines were maintained at 37°C in DMEM medium (Gibco®) supplemented with 10% FBS, 1% penicillin/streptomycin (Gibco®), 1% MEM non-essential amino acids (cellgro®) and 1% MEM vitamins (Gibco®) in 5% humidified CO_2_ chamber. All cell lines were determined to be free of mycoplasma using commertial PCR detection kit (MycoScope™, Genlantis, CA).

### Cell proliferation assay

Canine OSA cells were plated in triplicate at a density of 800 cells for Abrams and 2000 cells for Moresco and D17 in 96 well plates. Cells were cultured for at least 24 h prior to the experiment. Culture medium was replaced with fresh medium containing different concentrations of CPFX or ENFX at concentrations between 0–40 µg/ml for 4 days. Cell number was measured by incubation with the 3-(4,5-dimethylthiazol-2-yl)-5-(3-carboxymethoxyphenyl)-2-(4-sulfophenyl)-2*H*-tetrazolium, inner salt (MTS) one –solution assay reagent (CellTiter 96*, Promega) at 37°C for 1 h before reading absorbance on a spectrophotometer (SpectraMax 190, Molecular Devices) at 490 nm. CPFX and ENFX were tested in the absence of cells and determined not to interfere with tetrazolium salts. Independent assays were repeated a minimum of three times.

### Clonogenic assay

Cell survival was assessed by clonogenic assay as previously described [37]. In brief, cells were plated onto 6-well plates (1000 cells per dish) allowed to adhere overnight, and treated afterward with CPFX or ENFX at concentrations of 0, 1,5,10,20 and 40 µg/ml and incubated for 8 days. On day 8, the colonies were stained with crystal violet (in ethyl alcohol). Each experiment was repeated at least five times.

### Cell cycle Analysis

The effect of CPFX or ENFX on cell cycle distribution was determined by flow cytometry following staining of the cells with propidium iodide. Briefly, cells (2×10^5^ cells) were seeded and allowed to attach overnight. The medium was replaced with media without FBS and incubated for 30 h. Then, following 4 and 5 days of treatment with CPFX or ENFX at concentrations of 0, 1,5,10,20 and 40 µg/ml, floating and attached cells were harvested and centrifuged at 894× g for 3 min. The samples were washed with PBS and fixed in 70% ethanol. The cells were then treated with 100 µg/ml RNase A and 50 µg/ml propidium iodide for 30 min and analyzed using a FACScan flow cytometer and Cell Quest software (Becton Dickinson).

### Antibodies

Rabbit polyclonal anti-cdc2, mouse monoclonal anti-actin, and anti-p21 were purchased form Santa Cruz Biotechnology. Rabbit polyclonal anti-cleaved PARP, anti-cleaved caspase-3 and mouse monoclonal anti-phospho cdc25C was obtained from Cell Signaling .

### Western blot Analysis

After treatment with CPFX or ENFX at concentrations of 0, 20 and 40 µg/ml on Abrams for 4 and 5 days, floating and attached cells were collected and lysed. Lysed samples were heated at 95°C for 10 min and cleared by centrifugation at 10000× g for 20 min. Lysate protiens were resolved by sodium dodecyl sulfate-polyacrylamide gel electrophoresis (SDS-PAGE) and transferred onto nitrocellulose membrane. The membrane was incubated with Tris-buffered saline (PBS) containing Tween 20 (0.05%) and 3% (w/v) non-fat dry milk (PBST for phospho cdc25C) and exposed to the desired primary antibody at 4°C overnight. Following treatment with appropriate secondary antibody, the immunoreactive bands were visualized using enhanced chemiluminescence method.

### Caspase-3/7 activity assay

Apoptosis was assessed by measuring caspase-3/7 activity. In brief, 3×10^5^ Abrams cells were plated in 6-well plates in 2 ml of high glucose DMEM medium with 10% fetal bovine serum and incubated overnight at 37°C and 5% CO_2_. CPFX or ENFX (20 or 40 µg/ml) was added to the wells, and plates were incubated for an additional 72 h. Caspase-3/7 activity was then measured with a commercial assay performed in accordance with the manufacturer's specifications. Fluorescence was quantified with a spectrofluorometer microplate reader (SPCTRA max Gemini, Molecular Device) at an excitation wavelength of 354 nm and emission wave length of 442 nm. Cells treated with doxorubicin (0.5 µM) were used as the positive control, and untreated cells were used as the negative control. Samples were analyzed in triplicate, and each experiment was repeated 3 times.

### Statistical analysis

The results shown are representative of experiments repeated a minimum of three times. For MTS assay, the effects of CPFX and ENFX concentrations were compared individually for each drug and each cell line by one-way ANOVA and post-hoc Tukey's testing. For caspase 3/7 activity, one-way ANOVA and post-hoc Tukey's testing was applied to all groups. Differences were considered significant with a *p*<0.05.
